# Supplementary material for: An endothelial regulatory module links blood pressure regulation with elite athletic performance
Source: PLoS Genet. 2024 Jun 17;20(6):e1011285. doi: 10.1371/journal.pgen.1011285 (PMC11182536; doi:10.1371/journal.pgen.1011285)
Supplement: S6 Table — (DOCX) [file pgen.1011285.s006.docx]

**Supplementary Table S6**. The protein-coding genes that directly or indirectly interact with the regulatory region.

| **Human gene name** | **Nr of samples with interactions present** | **Interacting SNPs**  **(Chr 22)** | **Gene included in interaction figure** |
| --- | --- | --- | --- |
| RP4-614C15.2 | 13 | 46708982-6708983  46711134-6711135  46713281-6713282  46717741-6717742  46718112-6718113  46718963-6718964  46719795-6719796  46719905-46719906 | Yes |
| GNAS | 7 | 46708199-46708200  46711019-46711020  46714601-46715003  46715607-46715608  46718112-46718113  46719020-46719021 | Yes |
| SPO11 | 6 | 46711134-46711135  46713281-46713282  46718112-46718113  46719020-46719021 | Yes |
| PHACTR3 | 5 | 46708199-46708200  46711134-46711135  46714601-46715003  46718963-46718964 | Yes |
| ZNF831 | 5 | 46711134-46711135  46714601-46715003  46717741-46717742 | Yes |
| GNAS-AS1 | 4 | 46708982-46708983  46711134-46711135  46714601-46715003  46717741-46717742 | No |
| RP4-806M20.5 | 4 | 46708199-46708200  46711134-46711135  46718112-46718113  46718963-46718964 | No |
| ZBP1 | 4 | 46711134-46711135  46714601-46715003  46719020-46719021 | No |
| rs16982520 | 3 | 46714601-46715003  46715607-46715608  6718963-46718964 | No |
| TFAP2C | 2 | 46708199-46708200  46715607-46715608 | No |
| CDH26 | 1 | 46718112-46718113 | No |
| LINC00028 | 1 | 46713538-46713539 | No |
| WFDC10B | 1 | 46715607-46715608 | No |
| WFDC13 | 1 | 46717741-46717742 | No |
