# Supplementary material for: An endothelial regulatory module links blood pressure regulation with elite athletic performance
Source: PLoS Genet. 2024 Jun 17;20(6):e1011285. doi: 10.1371/journal.pgen.1011285 (PMC11182536; doi:10.1371/journal.pgen.1011285)
Supplement: S13 Table — (DOCX) [file pgen.1011285.s013.docx]

| **Breed** | **n** |
| --- | --- |
| Arabian horses | 29 |
| Thoroughbred | 30 |
| Standardbred | 45 |
| Coldblooded trotters | 210 |
| Finnhorses | 4 |
| Gotland pony | 2 |
| Shetland pony | 3 |
| Ardennes | 20 |
| Icelandic horses | 6 |
| North-Swedish draught | 18 |
| Exmoor pony | 23 |
| Fjord horse | 1 |
| Przewalski | 3 |

**Supplementary Table S13.** List of horses included in the MassArray genotyping
