# Supplementary figures and images for: An endothelial regulatory module links blood pressure regulation with elite athletic performance
Source: PLoS Genet. 2024 Jun 17;20(6):e1011285. doi: 10.1371/journal.pgen.1011285 (PMC11182536; doi:10.1371/journal.pgen.1011285)

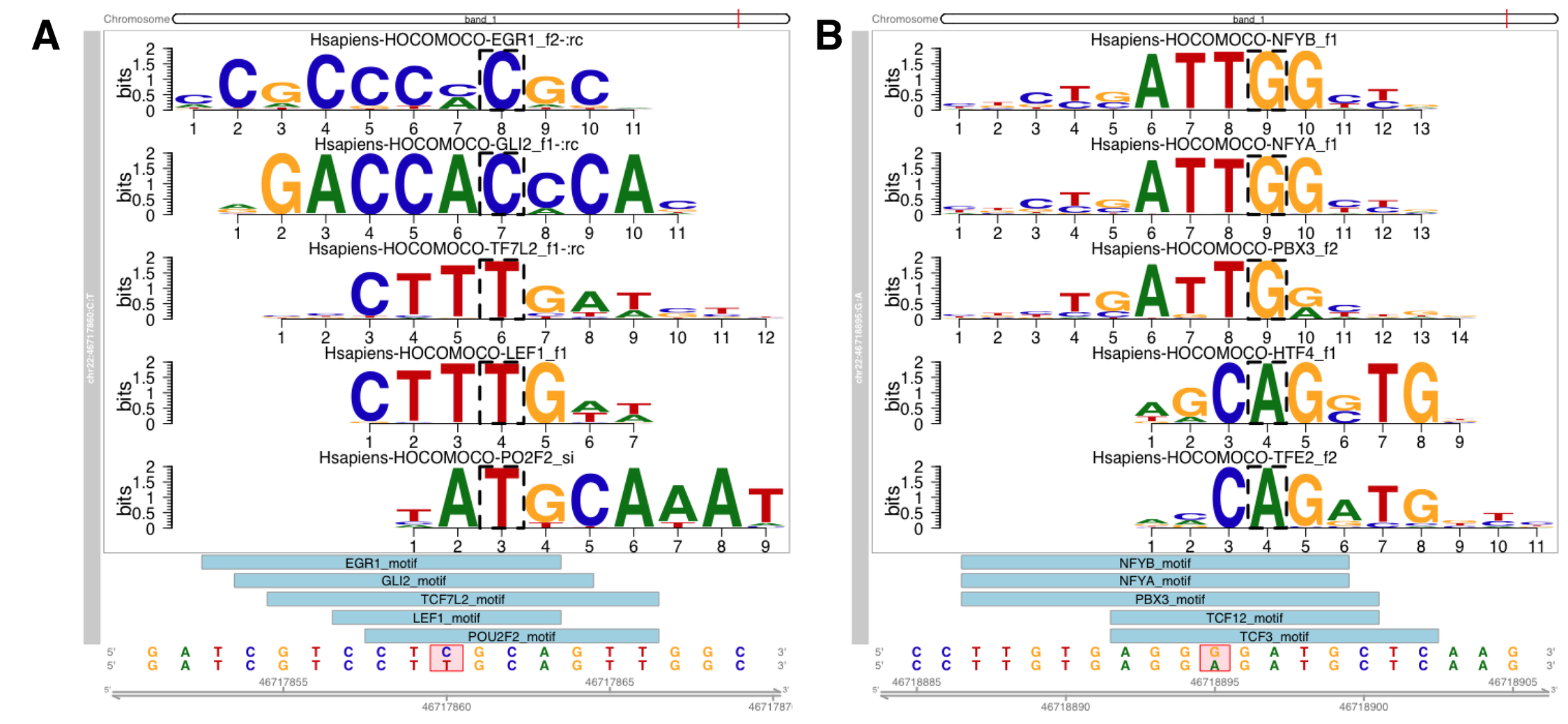

Supplement: S1 Fig — SNVs rs69244086 C>T (A) and rs69244089 T>C (G/A) (B) are illustrated. (TIF) [file pgen.1011285.s014.tif]
