# Supplementary material for: An endothelial regulatory module links blood pressure regulation with elite athletic performance
Source: PLoS Genet. 2024 Jun 17;20(6):e1011285. doi: 10.1371/journal.pgen.1011285 (PMC11182536; doi:10.1371/journal.pgen.1011285)
Supplement: Attachment — Submitted filename: CBTreview.pdf [file pgen.1011285.s015.pdf]

The manuscript, “An endothelial regulatory module links blood pressure regulation with elite athletic performance” outlines follow-up studies to investigate the possible role of a region of equine chromosome 23 in racing performance. The authors utilize data primarily from Coldblooded Trotters to evaluate how variation in this region may associate with blood pressure measured during exercise, plasma EDN3 levels, and race performance. To hypothesize how this variation may functionally differ they rely primarily upon data from human endothelial cells. They assert that these data support a likely enhancer in the region that alters the expression of genes associated with performance, studying an “elite” performance haplotype compared to a “subelite” haplotype and thus 2 groups of horses.

The study combines a great deal of information gathered from several sources. The most compelling of which I find to be a significant association of blood pressure during exercise with the haplotype deemed to represent “elite performers.” The authors have made a case that this region of the genome may contain variants that alter blood pressure and thus impact athletic performance. However, even though there is evidence of a regulatory function of this region in humans, little to no information is presented regarding the expression or regulation of this region of the genome in horses. I therefore believe the conclusions are overstated. Similarly, the title asserts that this study makes it certain that differences in genome regulation at this region alters blood pressure in the horse. I am not convinced that is the case given the data presented.

Additional lines of evidence of alterations in genome regulation in the horse are necessary to claim that these variants do alter expression of any/all of the candidate genes. Additional information and clarifications outlined below will also help strengthen the manuscript.

The model utilized is the Coldblooded Trotter (CBT), which had notable introgression from the Standardbred. The prior work identified a region of chromosome 23 in CBT that is hypothesized to have originated in Standardbreds, and that is thought to be associated with athletic performance.

The fact that the allele deemed to be favorable is at high frequencies in horses bred for racing compared to low frequency in ponies and draught horses is not necessarily support that it is associated with racing. There are many genomic regions that differ between these types of horses – some due to performance, but many that are likely due only to demographics, general breed type (e.g., light vs draft) and breed ancestry. Even so, it is not clear where this statement is originating as several pony samples in the study (Table 5) have a moderate frequency of the “elite” allele including the Shetland and Gotland pony (although N=2 in the latter)).

With the discussion of allele frequencies in other breeds, the statement on line 251-252 that the genotypes did NOT distribute according to HWE is false. The data in Table 5, and my own calculations given the allele frequencies presented, demonstrate that all samples except for the Finnhorse (N=4) adhere to the expectations of HWE. This result contradicts the entire premise that the locus is under selection in the CBT, especially if it were to have originated from the Standardbreds, in which the impact of gene flow would also throw off HWE expectations. As an aside, the calculation of a p-value for HWE for samples of 20 or fewer individuals (the case in many samples) is not informative.

As mentioned, the authors pose that this region was introgressed from the Standardbreds and selected for in the CBT. Given the high performance of Standardbreds, they then assert that variation in this region yields benefits to performance in these CBT. It is sensible to hypothesize that CBT with this introgressed region may not only have that part of their genome derived from Standardbreds, but also

may contain other regions that persist due to prior admixture. It would be beneficial to determine if the CBT horses with the elite performance haplotype have a greater proportion of Standardbred ancestry overall than the others.

Data from the equine FAANG project, which mapped regulatory regions of the genome of Thoroughbreds, was evaluated. They report finding no evidence of genome regulation in the data available (which do not include endothelial cells). The authors note that genome regulation is highly species-specific but then still rely upon the human data (HiCap from endothelial cells) to draw conclusions. A little troublesome, they also note (line 204) that the region is "not well conserved" between humans and horses; reporting the percent conservation would be beneficial. The authors therefore argue both that genome regulation is species-specific and that the region is not well conserved, but still base the entirety of the thesis of the manuscript on regulatory data from humans that they say indicates "regulatory potential" of the region in horses. As an aside, I looked at public tracks made available from the equine FAANG project and do see evidence of regulatory regions in this area. If this is indeed the case, this would greatly improve the hypotheses presented.

As noted earlier, the authors do a nice blood pressure study test of horses with each haplotype at rest, during, and post-exercise. Blood pressure does not differ by haplotype at rest but does during and post-exercise. This result is intriguing, and the authors make a case for the role of altered regulation of EDN1 and EDN3 in the elite haplotype horses as causative of this observation. Information that would lend further support to that result includes a description of the level of fitness/training of the horses studied, and a discussion of how sex might influence this measure (it appears (Sup Table 2) that all the horses from which exercise BP was measured that had the sub-elite haplotype were males while several females were in the elite performing group). The statistics utilized also need to be revisited as the authors talk about the effect of haplotype on blood pressure but also note a significant interaction between haplotype and time of measurement. Given a significant interaction, neither main effect should be discussed individually.

I disagree strongly with the claim that the "elite haplotype has an additive effect on performance traits" (line 240). This section is generally difficult to follow but both fail to find an association of haplotype on racing performance in the Standardbreds and instead of showing an additive effect, appears to show a possible dominant impact on several measures of racing success in the CBT. If this region was introgressed from the Standardbreds due to its functional effect, why is it not significant in those horses? In this section claiming an additive effect, the authors make a case for dominance in lines 246-247, making it puzzling why an additive effect was focused upon at all. The data used also needs to be clarified and possibly reconsidered. In Tables 3 and 4, number of starts, number of wins, earnings, etc. are reported. It is not clear if the number of wins, places, and earnings were corrected for the number of starts (# of wins can't be compared if # of starts differs as there are not equal opportunities to win). Further, both populations suffer from a relatively low samples of TT (subslite) individuals (only 7 in the case of the Standardbred). Would a contingency table be a more appropriate statistical test?

The subelite haplotype was found to be "borderline" significant with number of wins in SBs (again see sample size issue). Were all these Standardbreds trotters? Could pacers influence the population structure and interpretation of data?

Differences in plasma EDN1 and EDN3 concentrations are compelling given the discussion of what is known about their role in blood pressure regulation in humans. The authors, however, note 15 genes in the region that could be influnced by the putative regulatory region (line 226) and putative interactions

of the variation with other genes (GNAS, SPO11, ZNF831). It may be that EDN1 and EDN3 are the "causative" genes, but even if a regulatory region in the horse was definitively shown, these other genes have not been ruled out as important to the phenotype.

More minor clarifications:

- Methods (line 555) notes that WGS variants were retained if their genotyping pattern in pooled data was similar between CBT and SB and not in NSD." Please explain the pooled genotyping process. Could this approach have biased the analysis?
- Table 1. Clarify which haplotype(s) are considered elite and subelite
- It is not clear what the haplotype coefficient is in Table 2. What performance measure(s) are included?
- Table 3 – clarify in the legend that the C allele is tagging the elite performance group.
- Table 5 – why test HWE when N is so small? Only STBD and CBT are >30 samples. Those with 20 or more samples are in HWE!!! Why did we think do this? Line 251-252. They DO fit HWE. There is therefore no evidence of selection based upon frequencies.
- Line 241 claims the 14 variants in the 5.5kb region of interest were in perfect LD. Clarify that is only in CBT and Standardbreds (Table 1 shows other haplotypes in the other breeds evaluated).
- Blood pressure and plasma EDN were both associated with haplotype. Were these two measures correlated with one another?
- The discussion needs to be made more concise (the 2nd paragraph is nearly 3 pages long), and focus on what is known rather than speculation.
- The results of genotyping on other equids could be better explained/discussed.
- The assertion that this may also impact osteogenesis is weak (line 435) and should be removed.
