# Supplementary material for: An endothelial regulatory module links blood pressure regulation with elite athletic performance
Source: PLoS Genet. 2024 Jun 17;20(6):e1011285. doi: 10.1371/journal.pgen.1011285 (PMC11182536; doi:10.1371/journal.pgen.1011285)
Supplement: Attachment — Submitted filename: Response to Reviewers.docx [file pgen.1011285.s016.docx]

**Response to Reviewers**

Please note that the line numbers used in the response refer to the manuscript version without track changes.

Reviewer #1:

Fegraeus et al. report a follow-up study on earlier work regarding the genetics of athletic performance in horses. Starting from a previously identified and published sweep region of 19.6 kb downstream of the EDN3 gene, the authors were able to refine the associated region to 5.5 kb. This region harbors 14 single nucleotide variants (SNVs) that are in strong linkage equilibrium with each other. The authors determined that only two major haplotypes for these 14 SNVs segregate in the general horse population, which they termed elite performing haplotype (EPH) and subelite performing haplotype (SPH). Extrapolating from existing human data, Fegraeus et al. identify potential regulatory networks that provide new plausible and testable hypotheses how the variants might ultimately affect endothelin signalling and blood pressure regulation. The proposed phenotypic effect on endothelin serum levels and blood pressure was validated in horses with the different genotypes. Overall, this is a comprehensive and well presented study that enables a significantly improved understanding of the complex traits under investigation.

Specific comments:
(1) The data availability statement is inacceptable. The raw data must be made publicly available prior to the publication of this manuscript and accession numbers must be given in the manuscript. Individual phenotypes and marker genotype data of the studied horses must be given in one or more supplementary tables to allow interested readers to repeat the presented analyses.

Author response (1)

Thank you for pointing this out. We have added the genotype data in Supplementary file S12. We have also added the following information to the section “Availability of data”, in the manuscript. “The racing performance data that support the findings of this study are available from the Swedish Trotting Association (Stockholm, Sweden), but restrictions apply to the availability of these data, which were used under license for the current study, and so

are not publicly available. However, data are available from the authors upon

reasonable request and with permission of the contact person at the Swedish Trotting Association, Christina Olsson. The WGS data has been deposited in the BioProject database (NCBI repository SRA) with ID PRJNA1045044 according to their guidelines. According to the ethical permit related to the study (application number 2006/784-31/1 and 2012/1633-31/4) approved by the Human Research Ethics Committee at Karolinska Stockholm, Sweden, the sequencing data from living patients cannot be shared due to patient privacy regulations. We therefore only share the summary or processed form of the sequencing data in Supplementary tables S5-S7. More information can be found at Etikprövningsmyndigheten, Box 2110, 750 02, Uppsala, or [registrator@etikprovning.se](mailto:registrator@etikprovning.se).” All other data analyzed can be found in the manuscript or in supplementary files.

(2) Figure 1: The figure would be much easier to grasp, if the displayed species were indicated in each of the four panels A-D (horse or human). The authors may want to consider to redesign panel D in order to better display the correspondence between horse and human. Alternatively, another supplementary figure may be added to display more details of the horse-human correspondence (e.g. a dot plot and/or a full sequence alignment).

Author response (2)

Thank you for the suggestions. We have re-made the figure according to the reviewer´s suggestions.

(3) Can you add some additional text with more information why the human sequence is twice as long as the horse sequence (11 kb vs 5.5 kb)? What is the repeat content in the two species in this region?

Author response (3)

As displayed in figure 1, there is a gap in the horse sequence compared to the human sequence. As such, the difference in length between horse and human is not due to repeat content.

(4) Figure S2: Please expand the figure legend and give more explanations. The legend should contain an easy to grasp list/explanation of all transcription factors, for which binding site consensus sequences are shown. Is a score or any other kind of measure available, how likely it is that these transcription factor will bind to the reference and alternate alleles? A supplementary table with all TF binding scores for all analyzed variable sites should be added.

Author response (4)

Thank you for the comment. Supplementary Figure 2 shows the five most significant transcription factors for the two sites. We have clarified that in the legend. The names of the TFs are already included in the figure. We have also included a Supplementary Table S8, with TF binding scores for all analyzed variable sites, as requested.

(5) Consider to replace SNP with SNV.

Author response (5)

This has been amended throughout the manuscript

(6) I assume that all horse genomic coordinates in this study refer to the EquCab3.0 assembly. If true, please state this explicitly at the beginning of the methods section.

Author response (6)

Yes, we refer to the EquCab3.0 assembly throughout the manuscript. This is now stated in the beginning of the material and methods section, line 496.

(7) Line 266: "Data not shown" is not allowed in PLoS Genetics publications. All underlying raw data must be made publicly available.

Author response (7)

Thank you for pointing this out. We have removed this statement and instead added a reference, line 270.

(8) Line 389: Myostation -> myostatin

Author response (8)

Thank you for noticing this, this has been corrected

(9) Line 664: Suit --> Suite (?)

Author response (9)

Thanks for noticing, we have corrected this

Reviewer #2:

The authors describe the identification of a causative genomic region (5.5 kb/14 SNPs, regulatory element acting on EDN3 transcription) for blood pressure regulation and athletic performance in horses based on fine-mapping and comparative analyses. Then, the authors defined and identified an elite performing haplotype (EPH) and a sub-elite performing haplotype (SPH). EPH is associated with higher plasma levels of EDN3 and lower levels of EDN1, and lower exercise-related blood pressure.
This research is well organized, but the following revision or comment are necessary.

I have read this manuscript in a previous round of peer review. In the previous manuscript, it was unclear whether the 5.5 kb site truly functioned as an enhancer site, as there were no results from gene expression analysis. The new Human HiCap analysis performed in this study may have compensated for previous weaknesses.

However, this reviewer has some concerns about Human HiCap analysis. While the author's main research is conducted on horses, HiCap analysis is conducted on humans. Is it appropriate to conduct an analysis using cells from different species in this way? Has this approach been used in other studies? So, the authors should discuss discrepancies with horse FAANG and human HiCap analyses.

Author response
Our across-species strategy is based on the fact that it has been shown multiple times that the expression profiles of similar tissues across different species are more correlated than those of different tissues from the same species (eg. Merkin, J., Russell, C., Chen, P. & Burge, C.B. *Science* 338, 1593-9 (2012), Brawand, D. *et al.* *Nature* 478, 343-8 (2011), Chan, E.T. *et al.* *J Biol* 8, 33 (2009)). For example, it is the different expression patterns of the identical master transcription factors in human and chimpanzee brains that produce developmental and perhaps cognitive differences between the two species (Nowick, K., Gernat, T., Almaas, E. & Stubbs, L. *Proc Natl Acad Sci U S A* 106, 22358-63, 2009).

Yes, it has been shown that HiCap can pinpoint a level of conservation in the regulatory machinery across species, for example, please see: Sylvain Foissac, Sarah Djebali, Kylie Munyard, Nathalie Vialaneix, Andrea Rau, et al., Multi-species annotation of transcriptome and chromatin structure in domesticated animals. BMC Biology, 2019, 17 (1), 25 p. 10.1186/s12915-019-0726-5. hal-02437599. We recommend the reviewer scrutinize Figure 8: “The significant and systematic difference of gene expression and chromatin accessibility values between A and B compartments (*p* values < 2.2 × 10^−16^ overall, Wilcoxon tests) confirms a general consistency between RNA-seq, ATAC-seq and Hi-C data across species”.

Using HiCap from humans simply supports that this region has a regulatory effect. The results make sense since blood pressure genes are targeted, as expected. HiCap using horse cells will be done in the future when we receive more funding for this project. The reviewer is correct in that horse cells are better than human cells. However, the use of human cells in the HiCap experiment identifies novel chromatin interactions using this region, that in our opinion adds significant comparative value to this study.

Using updated FAANG data, we indeed see that there is an active enhancer in lamina from horse FAANG histone mark data, so we do not see any discrepancies between equine FAANG and the human HiCap data.

The author conducted WGS to comprehensively identify variants, but the number of animals analyzed seems to be small. Is there a possibility that a causative variant exists at a site distant from the 5.5 kb site?

Author response

We agree with the reviewer that the number of horses with WGS data is small, but since we are contrasting the different phenotypes and haplotypes, these animals must carry the causative variant(s), this is a sure thing! When we identified the DMRT3 stop mutation (Lisa Andersson et al, Nature 2012) we analyzed two by two WGS horses, so this strategy has worked well for us previously.

Here we have tested SNPs within and outside of the haplotype on chromosome 22 for association with racing performance traits, and there are no significant associations between any of the SNPs outside of our identified haplotype and performance traits. The basis for defining our haplotype is whether or not the SNPs associates with performance or not, hence the causative mutation must reside within the haplotype for this particular trait. Modifiers are of course always possible to be present, fine tuning the traits. The identification of such modifiers will likely be obtained in future studies, just like we recently have done for DMRT3 (a revised manuscript for GSE).

Reviewer #3:

The manuscript, “An endothelial regulatory module links blood pressure regulation with elite athletic performance” outlines follow-up studies to investigate the possible role of a region of equine chromosome 23 in racing performance. The authors utilize data primarily from Coldblooded Trotters to evaluate how variation in this region may associate with blood pressure measured during exercise, plasma EDN3 levels, and race performance. To hypothesize how this variation may functionally differ they rely primarily upon data from human endothelial cells. They assert that these data support a likely enhancer in the region that alters the expression of genes associated with performance, studying an “elite” performance haplotype compared to a “subelite” haplotype and thus 2 groups of horses.

The study combines a great deal of information gathered from several sources. The most compelling of which I find to be a significant association of blood pressure during exercise with the haplotype deemed to represent “elite performers.” The authors have made a case that this region of the genome may contain variants that alter blood pressure and thus impact athletic performance. However, even though there is evidence of a regulatory function of this region in humans, little to no information is presented regarding the expression or regulation of this region of the genome in horses. I therefore believe the

conclusions are overstated. Similarly, the little asserts that this study makes it certain that differences in genome regulation at this region alters blood pressure in the horse. I am not convinced that is the case given the data presented.

Author response

Thank you for this comment. Although our conclusions are based on HiCap data from humans, we believe that our racing performance results and blood pressure measurements in horses supports this theory. We have been careful to state in the manuscript that this is a potential regulatory region and that it may affect blood pressure in horses, e.g., line 313. We have also added the word potential to the conclusion, to emphasize the fact that we do not have complete evidence for the function in horses, line 467.

Additional lines of evidence of alterations in genome regulation in the horse are necessary to claim that these variants do alter expression of any/all of the candidate genes. Additional information and clarifications outlined below will also help strengthen the manuscript.

Author response

We agree with the reviewer that more data and information from horses would strengthen the manuscript. However, we have been very careful to not claim that these variants alter the expression of any genes but we believe that there is a potential regulatory region, given the phenotypic differences we observe in the horses.

The model utilized is the Coldblooded Trotter (CBT), which had notable introgression from the Standardbred. The prior work identified a region of chromosome 23 in CBT that is hypothesized to have originated in Standardbreds, and that is thought to be associated with athletic performance.

The fact that the allele deemed to be favorable is at high frequencies in horses bred for racing compared to low frequency in ponies and draught horses is not necessarily support that it is associated with racing. There are many genomic regions that differ between these types of horses – some due to performance, but many that are likely due only to demographics, general breed type (e.g., light vs draft) and breed ancestry. Even so, it is not clear where this statement is originating as several pony samples in the study (Table 5) have a moderate frequency of the “elite” allele including the Shetland and Gotland pony (although N=2 in the latter)).

Author response

We fully agree with the reviewer that many genomic regions differ between horse breeds, and obviously not all of them are related to performance. However, in this study, we have tested all SNPs on chromosome 22 for association with racing performance traits.

We found that the association to these traits drops significantly outside of our identified haplotype. We also note a significant difference in frequency of the alleles, when comparing breeds traditionally used for racing, with breeds used for agricultural work and pleasure riding. We argue that this is evidence that this genomic region is important for racing performance. Similar strategies have been used in other horse studies, i.e. the identification of the DMRT3 gene as being crucial for horses to perform ambling gaits (Lisa Andersson et al. Nature, 2012), where the frequency of the alternate allele was high in all breeds with the ability to perform ambling gaits, and low or absent in three-gaited breeds.

As the reviewer pointed out, some pony breeds had a moderate frequency of the alternate allele. In Sweden, the Shetland pony and Gotlandsruss are used both for pleasure riding and driving but also for harness racing competitions. The horses analyzed in the current study include both types and the allele frequencies may reflect this.

With the discussion of allele frequencies in other breeds, the statement on line 251-252 that the genotypes did NOT distribute according to HWE is false. The data in Table 5, and my own calculations given the allele frequencies presented, demonstrate that all samples except for the Finnhorse (N=4) adhere to the expectations of HWE. This result contradicts the entire premise that the locus is under selection in the CBT, especially if it were to have originated from the Standardbreds, in which the impact of gene flow would also throw off HWE expectations. As an aside, the calculation of a p-value for HWE for samples of 20 or fewer individuals (the case in many samples) is not informative.

Author response

Thank you for the comments. We understand the reviewer's concern regarding the HWE calculations and sample number. As such, we have removed the calculations and p-values for HWE for all horse breeds except for the Standardbred and Coldblooded trotters. As some breeds were fixed for one allele, the HWE calculation is not reliable. However, the fact that one allele is fixed could be an indication that there has been selection for this variant in the specific breed. But as the reviewer pointed out, the Coldblooded trotters and Standardbreds are in HWE, and this has been corrected in the manuscript, line 257-258.

As mentioned, the authors pose that this region was introgressed from the Standardbreds and selected for in the CBT. Given the high performance of Standardbreds, they then assert that variation in this region yields benefits to performance in these CBT. It is sensible to hypothesize that CBT with this introgressed region may not only have that part of their genome derived from Standardbreds, but also may contain other regions that persist due to prior admixture. It would be beneficial to determine if the CBT horses with the elite performance haplotype have a greater proportion of Standardbred ancestry overall than the others.

Author response

Thank you for the comments. We agree that it would be highly informative and beneficial to investigate that. However, unfortunately we do not have enough information and data to perform such analysis.

Data from the equine FAANG project, which mapped regulatory regions of the genome of

Thoroughbreds, was evaluated. They report finding no evidence of genome regulation in the data available (which do not include endothelial cells). The authors note that genome regulation is highly species-specific but then still rely upon the human data (HiCap from endothelial cells) to draw conclusions. A little troublesome, they also note (line 204) that the region is “not well conserved” between humans and horses; reporting the percent conservation would be beneficial. The authors therefore argue both that genome regulation is species-specific and that the region is not well conserved, but still base the entirety of the thesis of the manuscript on regulatory data from humans that they say indicates “regulatory potential” of the region in horses. As an aside, I looked at public tracks made available from the equine FAANG project and do see evidence of regulatory regions in this area. If this is indeed the case, this would greatly improve the hypotheses presented.

Author response

Please also see response above. Yes, gene regulation is often species specific, but an overall conservation of the regulatory machinery is likely to exist. For example, our regulatory region was pinpointed in the Zoonomia project article by Sullivan P et al., Leveraging base-pair mammalian constraint to understand genetic variation and human disease. 2023 Apr 28;380(6643):eabn2937. doi: 10.1126/science.abn2937. Epub 2023 Apr 28. It is not surprising that this region has a primate-specific insertion, this is rather expected.

As noted earlier, the authors do a nice blood pressure study test of horses with each haplotype at rest, during, and post-exercise. Blood pressure does not differ by haplotype at rest but does during and postexercise. This result is intriguing, and the authors make a case for the role of altered regulation of EDN1 and EDN3 in the elite haplotype horses as causative of this observation. Information that would lend further support to that result includes a description of the level of fitness/training of the horses studied, and a discussion of how sex might influence this measure (it appears (Sup Table 2) that all the horses

from which exercise BP was measured that had the sub-elite haplotype were males while several females were in the elite performing group). The statistics utilized also need to be revisited as the authors talk about the effect of haplotype on blood pressure but also note a significant interaction between haplotype and time of measurement. Given a significant interaction, neither main effect should be discussed individually.

Author response

Thank you for the relevant comment. All horses, except for the 2-year old horses, were in competing condition, which we have stated in the material and methods (line 726). For the blood pressure analysis, all horses but five were males. Only two of the females had blood pressure data during exercise and both horses carried the EPH haplotype. We have re-run the blood pressure analyses during exercise, and included sex as a factor in the model. There were no significant effects of sex on any of the blood pressure parameters. Regarding the significant interaction between haplotype and time of measurement, the per variable effects are less reliable than the total sum, and so it is the sum that will be discussed because of the interaction effect.

I disagree strongly with the claim that the “elite haplotype has an additive effect on performance traits” (line 240). This section is generally difficult to follow but both fail to find an association of haplotype on racing performance in the Standardbreds and instead of showing an additive effect, appears to show a possible dominant impact on several measures of racing success in the CBT. If this region was introgressed from the Standardbreds due to its functional effect, why is it not significant in those horses? In this section claiming an additive effect, the authors make a case for dominance in lines 246-

247, making it puzzling why an additive effect was focused upon at all. The data used also needs to be clarified and possibly reconsidered. In Tables 3 and 4, number of starts, number of wins, earnings, etc. are reported. It is not clear if the number of wins, places, and earnings were corrected for the number of starts (# of wins can’t be compared if # of starts differs as there are not equal opportunities to win). Further, both populations suffer from a relatively low samples of TT (subslite) individuals (only 7 in the case of the Standardbred). Would a contingency table be a more appropriate statistical test?

Author response

Thank you for pointing this out. We agree with the reviewer that the statement that the elite haplotype has an additive effect on performance traits may not be completely accurate, this has been corrected in the manuscript, line 244.

Our results from the racing performance analyses are similar to previous studies on DMRT3, where the effect of the specific SNP differ between Standardbreds (SBs) and Coldblooded trotters (CBTs) (i.e. <https://doi.org/10.2527/jas.2014-7803> and <https://doi.org/10.1038/nature11399>). As SBs have been selected for racing performance for a much longer time period, compared to the CBTs, most SB horses carry the variant allele, and therefore its effect on performance are not as obvious as for the CBTs. Also, the fact that the allele frequency in the North-Swedish draft horses is significantly different from the frequency in CBTs, indicates that this region is of importance for racing performance.

For the racing performance analyses, in Table 3 and 4, we have table footnotes stating: “Sex, age, birth country, number of starts and *DMRT3* genotypes were included as fixed effects (when significant)”. Number of wins, places and earnings were all corrected for the number of starts, when significant.

We agree with the reviewer that the small number of SBs with the TT genotype will influence the results, and all statistical models would be susceptible to a small number of TT individuals, which is also observed here as the effect of the SB TTs is only seen when the TT genotype is incorporated into the CC/TT+TC test. Our results are very similar to the results observed for the DMRT3 mutation, where the majority of SBs are homozygous for the A-variant, while the frequency of the C-variant is much higher in the CBT population. Also, for DMRT3 the effect of the mutation differs between the two breeds (see Jäderkvist et al., 2014, The DMRT3 ‘Gait keeper’ mutation affects performance of Nordic

and Standardbred trotters). We appreciate the reviewer´s suggestion to analyze the data with another test, but, after discussing with our statistical advisor we do not believe that this will have any major impact on our results, as all statistical tests will be impacted by the low number of TT SBs. Ideally, we would like to increase the number of horses, and especially the number of TT horses analyzed. However, here we have tried to include as many TT horses as possible, meaning that the frequency of TT Standardbreds in the whole population is likely smaller than in our data, making it difficult to find enough TT horses for the analysis.

The subelite haplotype was found to be “borderline” significant with number of wins in SBs (again see sample size issue). Were all these Standardbreds trotters? Could pacers influence the population structure and interpretation of data?

Author response

All of the horses are Standardbred trotters. In Sweden only trotters are allowed to compete, there are no pace races and no pacers are included in our data.

Differences in plasma EDN1 and EDN3 concentrations are compelling given the discussion of what is known about their role in blood pressure regulate on in humans. The authors, however, note 15 genes in the region that could be influnced by the putative regulatory region (line 226) and putative interactions of the variation with other genes (GNAS, SPO11, ZNF831). It may be that EDN1 and EDN3 are the “causative” genes, but even if a regulatory region in the horse was definitively shown, these other genes have not been ruled out as important to the phenotype.

Author response

We agree with the reviewer that we cannot completely rule out the surrounding genes as being causative. However, we believe that the EDN3 is a strong candidate gene as it is known to be involved in blood pressure regulation and we observe altered levels of the protein when comparing horses with different haplotypes. Having said this, it is likely that multiple genes co-regulate this specific complex trait, and we already have a paragraph in the discussion regarding multiple genes that may be involved in the regulation of blood pressure, line 346.

More minor clarifications:

- Methods (line 555) notes that WGS variants were retained if their genotyping pattern in pooled data was similar between CBT and SB and not in NSD.” Please explain the pooled genotyping process. Could this approach have biased the analysis?

Author response

The purpose of the study in CBT, SB and NSD was to identify genomic regions of importance for athletic performance in trotters, so the SNP pruning procedure should enrich for SNPs of importance for performance. Since the selected and tested SNPs indeed showed significant association with athletic performance, our pruning of SNP (and initial hypothesis) seems to make sense. We simply looked if there were alleles segregating in the pools of both trotter breeds that were not present in the draft horse pool.

- Table 1. Clarify which haplotype(s) are considered elite and subelite

Author response

The SPH and EPH concepts are defined in the text after Table 1. We believe that it would be confusing for the reader to include the information in Table 1.

- It is not clear what the haplotype coefficient is in Table 2. What performance measure(s) are included?

Author response

Haplotype coefficient, or effect size, is the effect of each haplotype compared to the base haplotype (H1) for each performance trait, i.e., one copy of the H2 haplotype will reduce the square root transformed number of starts with 0.93 compared to H1. If you would plot box plots of these H1 vs H2 values for each trait, you would see these differences.

- Table 3 – clarify in the legend that the C allele is tagging the elite performance group.

Author response

This has been corrected

- Table 5 – why test HWE when N is so small? Only STBD and CBT are >30 samples. Those with 20 or more samples are in HWE!!! Why did we think do this? Line 251-252. They DO fit HWE. There is therefore no evidence of selection based upon frequencies.

Author response

See response on previous comment about HWE. We have removed all HWE calculations for all breeds except SBs and CBTs.

- Line 241 claims the 14 variants in the 5.5kb region of interest were in perfect LD. Clarify that is only in CBT and Standardbreds (Table 1 shows other haplotypes in the other breeds evaluated).

Author response

This has been corrected.

- Blood pressure and plasma EDN were both associated with haplotype. Were these two

measures correlated with one another?

Author response

There were only 11 horses (4 SPH and 7 EPH) where we had information about both blood pressure and plasma EDN. When analyzing the correlation between blood pressure and EDN3 over all time points, there were no significant correlations. However, when only analyzing the values during exercise there was a significant correlation between EDN3 and DBP (diastolic blood pressure) and borderline significant correlation between MAP (mean arterial pressure) (P=0.07) and EDN3. There were also significant correlations between EDN1 and DBP, SBP as well as MAP, during exercise. This has been added to the manuscript, line 295.

- The discussion needs to be made more concise (the 2nd paragraph is nearly 3 pages long), and focus on what is known rather than specula􀆟on.

Author response

We do not agree with the reviewer since the 2^nd^ paragraph is only 114 words long, and the total length of the discussion is not particularly long either. In the discussion the authors should interpret their results and we believe that this is what we are doing.

- The results of genotyping on other equids could be better explained/discussed.

Author response

Genotyping of other equids can give insights into the ancestral state of a haplotype. That is why we genotyped such species too.

- The assertion that this may also impact osteogenesis is weak (line 435) and should be removed.

Author response

We agree and the sentence has been removed
