# Supplementary material for: An endothelial regulatory module links blood pressure regulation with elite athletic performance
Source: PLoS Genet. 2024 Jun 17;20(6):e1011285. doi: 10.1371/journal.pgen.1011285 (PMC11182536; doi:10.1371/journal.pgen.1011285)
Supplement: Attachment — Submitted filename: EDN_revision_review.docx [file pgen.1011285.s017.docx]

The manuscript is much improved. The authors have addressed most of my concerns and it is appreciated that they have corrected errors and stepped back on some assertions that, in my opinion, did not have significant backing. The addition of new regulatory data from the horse also helps support their thesis. Finally, as stated in the first review, the relatively novel phenotyping is a notable and important part of this study and the results do support the role of this region in blood pressure.

With respect to my prior comments and the response:

- The line of evidence that what was done in the DRMT3 paper justifies methods in this investigation gets tiresome. I agree that sometimes that method can yield interesting results but it still is important to address assumptions and possible issues with the method.
- I still maintain that it is possible to examine if the CBT with better performance also have more SB ancestry than others. The authors used SNP chip data from over 600 CBT (referenced in line 491) and although I don’t see N for SB, I assume the dataset is similarly large. An admixture or structure analysis could quickly result in estimates of proportion of assignment of the CBT to the SB breed and help address this question.
- My comment about information on the other equids was not clear. The intent was that information on what data were used (accession numbers) needs to be given.

New comments/critiques:

The evidence that the region of EDN3 is possibly harboring a regulatory element for blood pressure is now more convincing. I still have some issues with the authors arguing both that regulatory elements can operate “over long genomic distances” (line 128) and that the putative regulatory elements identified are important because of their proximity to the loci of interest. The evidence reported supports endothelins as functional with respect to the phenotype but the authors do note other genes (15) in the region that are likely to be interacting with the regulatory elements. The discussion focuses on the endothelin results as expected but also spends quite a bit of time on other genes. I do not disagree (as stated above) that these genes may be important to performance phenotypes. It seems that the title should then be changed to recognize that the result is broader than just endothelin. Similarly, the authors then bring up other roles of endothelin in addition to blood pressure regulation, which is also a centerpiece of the title. All that said, the title could probably better reflect the results.

Additional comments:

It would be helpful if chromosome identities could be used instead of (or in addition to) the contig IDs. This just helps readability and other researchers more easily identify the genomic region of interest. Similarly, although at one point it is noted that the region of interest is on chr22, please provide the proper SNP identifiers (chr g. pos SNP) in the text (for instance line 154), and in the tables/supplementary tables.

The HOCOMOCO analysis is a little unclear. In addition to the variant loci, what is the similarity of other loci within the motif between horses and humans? (Sup Fig 1). I don’t argue that some of the loci may be very relevant, it is just difficult to believe that ALL of them are predicted to have high effects or a “strong” binding score.

I suggest stating the interaction effect (line 282) prior to the results of post-hoc tests for timepoints and haplotype.

Given a threshold set, a result is either significant or not. Please update line 296 to remove “borderline significant.” It was not significant given the threshold set – you can still report the p-value for readers to see and draw their own conclusion.

Methods – line 548, clarify what is the “required segregation pattern.”

It is well accepted that the DMRT3 genotype is significant to racing performance. However, as shown in the Supplement, 92 of CBT did not have a DMRT3 genotype. How was this handled? It seems quite important to the result.

Describe the method (software and parameters) used to phase haplotypes (line 599).

The 14 fine-map variants (as shown in Fig 1B) appear to be quite biased toward the distal end of the 5.5kb region of interest (10 of the 14 within 2500bp). Could the lack of representation of markers earlier in the region be biasing results? With the marker distribution it is not surprising that these markers are in high LD.

Is this a typo on line 690: “and not included as standard?” This needs correction/clarification.
