# Supplementary material for: An endothelial regulatory module links blood pressure regulation with elite athletic performance
Source: PLoS Genet. 2024 Jun 17;20(6):e1011285. doi: 10.1371/journal.pgen.1011285 (PMC11182536; doi:10.1371/journal.pgen.1011285)
Supplement: Attachment — Submitted filename: Response to reviewers.docx [file pgen.1011285.s018.docx]

Please note that the line numbers in the text refer to the manuscript version without track changes.

**Comments to the Authors:
Please note here if the review is uploaded as an attachment.**

Reviewer #1: The authors have adressed my comments satisfactorily.

The data availability statement is now acceptable to me. I find it regrettable that the ethics permit for this study was issued by a Human Research Ethics Committee rather than a committee related to animal welfare (an IACUC). This study does not relate to human patients, it relates to animals. Furthermore, it is my understanding that this study is about variation of physiological blood pressure parameters, not a disease. I therefore think that the term "living patient" in the data availability statement is inappropriate. I do not understand why sequencing data of animals cannot be made publicly available. This sets a very problematic precedence for future research involving animals. However, I assume that it is not in the power of the authors to change legal requirements in Sweden.

Author response

We think there has been a miscommunication between reading the sections, “Ethics approval and consent to participate” and “Availability of data and materials”. We have separate Swedish ethical approvals for both the human and horse studies included in this manuscript. We note in the manuscript under “Ethics approval and consent to participate” that horse sample collection approval was granted by an animal board in Uppsala and human sample collection was granted by a human ethics committee in Solna (Karolinska Institute).

In “Availability of data and materials”, we state how horse and human phenotypes and genotypes can be accessed. For the horse sequencing data, we have shared this data in SRA (PRJNA1045044), but due to commercial sensitivities associated with phenotypes, i.e., horse racing performance traits, we cannot link the phenotypes to the genotypes. We do state that the phenotypes can be requested, “authors upon reasonable request and with permission of the contact person at the Swedish Trotting Association, Christina Olsson.”

The remainder of the section refers to the protected sharing of human phenotypes and genotypes as enforced by the human ethics committee in Solna (Karolinska Institute).

Reviewer #2: This study comprehensively identified about 5.5 kb-region as endothelial regulatory module involved in blood pressure control based on racehorse exercise performance and blood pressure measurements, and the human HiCap data and transcription factor analysis.

This manuscript has been revised multiple times, with revisions noted by this reviewer. Therefore, this reviewer considers the current manuscript to be suitable for publication.

However, this reviewer requests comments or revisions on the following:

Comments:
In this study, elite haplotypes showed a high frequency in CBT and SB, but a low frequency of 0.07 in TB. Since TB is a racehorse, it may be expected that the frequency of this haplotype would be higher as the identified region is associated with racing ability. Why isn't that the case? Is it due to the difference in thoroughbred and trotter racing? Or does this allele not function significantly in thoroughbreds? This reviewer was concerned because the title stated "with elite athletic performance."

Author response

We think there has been a miscommunication. Table 1 reports that the elite performing haplotype (EPH, AAGCGTTTCTCAAA) is found in high frequency in both CBT, SB and Thoroughbreds. The frequency is 0.93 in the Thoroughbreds. The 0.07 value refers to the frequency of the sub-elite performing haplotype (SPH). We have double checked the text for an inversion of these numbers, but cannot find an example. If we have missed this, we ask the reviewer for clarification.

Line 389: myostatin?

Author response

Thanks for noticing. We have corrected this.

Reviewer #3: please see attached

The manuscript is much improved. The authors have addressed most of my concerns and it is appreciated that they have corrected errors and stepped back on some assertions that, in my opinion, did not have significant backing. The addition of new regulatory data from the horse also helps support their thesis. Finally, as stated in the first review, the relatively novel phenotyping is a notable and important part of this study and the results do support the role of this region in blood pressure.

With respect to my prior comments and the response:

- The line of evidence that what was done in the DRMT3 paper justifies methods in this investigation gets tiresome. I agree that sometimes that method can yield interesting results but it still is important to address assumptions and possible issues with the method.
- I still maintain that it is possible to examine if the CBT with better performance also have more SB ancestry than others. The authors used SNP chip data from over 600 CBT (referenced in line 491) and although I don’t see N for SB, I assume the dataset is similarly large. An admixture or structure analysis could quickly result in estimates of proportion of assignment of the CBT to the SB breed and help address this question.

Author response

We agree with the reviewer that this is an intriguing hypothesis, but it is not the aim of this manuscript. The goal of our current work was to refine the extent of the introgressed selective sweep, and to further investigate the functional impact of this region. We are not in a position to address the question of overall SB ancestry in CBTs and so test the reviewer’s hypothesis.

We do not have the dataset the reviewer assumes to test for global or, what would be better, local genetic ancestry. We have access to 4 SB WGS, 0 SB with 670 Array genotypes, and 629 CBTs with 670 Array genotypes. The 4 SBs do not represent enough genetic diversity for an accurate admixture analysis of the 629 CBTs.

For the purposes of this response, we did perform an admixture analysis using the 50K SNP chip data from our 2018 selective sweep paper [Jäderkvist Fegraeus K, *et al*. A potential regulatory region near the EDN3 gene may control both harness racing performance and coat color variation in horses. Physiol Rep. 2018 May;6(10):e13700. doi: 10.14814/phy2.13700]. This data set includes 42 horses (11 CBTs, 19 North-Swedish Draught horses, and 12 SBs). Again, this data is not ideal, as it does not include other breeds, and so can only test a three-way hypothesis. After performing quality control (Plink v1.9: --geno 0.2, --mind 0.2, --maf 0.05) we were left with 44,332 markers for analysis with Admixture v1.3. After cross-validation for *K*, K=3 was found to be the optimal solution. For the 11 CBTs assessed, the average SB admixture was 0.03 (SD 0.05), with a range of 0.00001-0.11683 (median = 0.00001). This is likely to be refined with both additional breeds to control for cryptic ancestry, and denser data panels.

- My comment about information on the other equids was not clear. The intent was that information on what data were used (accession numbers) needs to be given.

Author response

We are not sure what the reviewer means by this comment or what amendments are being requested. We suspect that there may have been confusion across the rounds of text revision. The only *other equids* we have included are Przewalski horses. This notation was corrected during the revision process. These are in-house Przewalski horse samples, and were not downloaded from SRA. We do note that the genotypes for these samples were missing from Supplementary Table 12, which we have now updated to now include all genotyped equid material, including the Przewalskis.

New comments/critiques:

The evidence that the region of EDN3 is possibly harboring a regulatory element for blood pressure is now more convincing. I still have some issues with the authors arguing both that regulatory elements can operate “over long genomic distances” (line 128) and that the putative regulatory elements identified are important because of their proximity to the loci of interest. The evidence reported supports endothelins as functional with respect to the phenotype but the authors do note other genes (15) in the region that are likely to be interacting with the regulatory elements. The discussion focuses on the endothelin results as expected but also spends quite a bit of time on other genes. I do not disagree (as stated above) that these genes may be important to performance phenotypes. It seems that the title should then be changed to recognize that the result is broader than just endothelin. Similarly, the authors then bring up other roles of endothelin in addition to blood pressure regulation, which is also a centerpiece of the title. All that said, the title could probably better reflect the results.

Author response

We appreciate the critiques and comments from this reviewer and during the review process we have adjusted our title accordingly. Our current title is: “An endothelial regulatory module links blood pressure regulation with elite athletic performance”. We feel that this title reflects the functional results from the endothelial tissue and the blood pressure measures we have made. We do not mention the gene *endothelin3.* It is probable that the module has effects across other tissues, but we do not want to overstate our results.

Additional comments:

It would be helpful if chromosome identities could be used instead of (or in addition to) the contig IDs. This just helps readability and other researchers more easily identify the genomic region of interest. Similarly, although at one point it is noted that the region of interest is on chr22, please provide the proper SNP identifiers (chr g. pos SNP) in the text (for instance line 154), and in the tables/supplementary tables.

Author response

During the review process of the manuscript, rs numbers (EVA version 4) became available for these SNVs. As rs numbers are unambiguous, unique and traceable through genome builds, unlike single nucleotide positions, we believe that these identifiers are the most valuable for the community. We have updated the manuscript accordingly.

Author response

The HOCOMOCO analysis is a little unclear. In addition to the variant loci, what is the similarity of other loci within the motif between horses and humans? (Sup Fig 1). I don’t argue that some of the loci may be very relevant, it is just difficult to believe that ALL of them are predicted to have high effects or a “strong” binding score.

To aid clarification, we have updated the methods to now state, “We assessed the impact of allele difference between the EPH and SPH haplotypes with hocomoco motifs (33), default position weight matrix to score each, at threshold of 1x10^-4^, before retaining the strong category of effects.”

As per the package, we use the supplied hocomoco database with 1,066 frequency matrices.

We would like to note that within the manuscript, we do not focus on the transcription factors themselves, rather the comparative transcription factor binding potential of the region. Comparative genomics can offer hints about function, but in the end functional proof is required. Recently, the cross-species conservation of transcription factor binding sites was addressed using the Zoonomia whole genome sequence alignment of 240 species (241 genomes, including equCab2 assembly and hg38) [Andrews et al., Mammalian evolution of human cis-regulatory elements and transcription factor binding sites. Science380, eabn7930 (2023). DOI:10.1126/science.abn7930]. That dataset spans 100 million years of evolution, and is perhaps best placed to address TFBS conservation in the absence of more empirical data. Andrews et al., analyzed 367 transcription human factors (15.6 million TFBSs) generated as part of ENDODE, and found that 2 million TFBSs are under evolutionary constraint in mammals. While this only represents ~13% of the sites measured, it still illustrates the utility of comparative analyses in hypothesis building.

I suggest stating the interaction effect (line 282) prior to the results of post-hoc tests for timepoints and haplotype.

Author response

Thanks for pointing this out, we have moved the sentence as suggested.

Given a threshold set, a result is either significant or not. Please update line 296 to remove “borderline significant.” It was not significant given the threshold set – you can still report the p-value for readers to see and draw their own conclusion.

Author response

We don´t want to overstate our results. We understand the reviewer´s point and see that the term borderline is not required. We have updated the text.

Methods – line 548, clarify what is the “required segregation pattern.”

Author response

Thank you for the comment. We have updated the manuscript to place the segregation pattern in the context of the 2018 paper (Jäderkvist Fegraeus K, *et al*. A potential regulatory region near the EDN3 gene may control both harness racing performance and coat color variation in horses. Physiol Rep. 2018 May;6(10):e13700. doi: 10.14814/phy2.13700). “From the SVs, only a 400 bp deletion (ECA22:46,714,602-46,715,003) matched the required segregation pattern of present in CBTs and SBs, reflective of a selection on crossbreed admixture (Jäderkvist Fegraeus K, *et al*.). The 400 bp deletion was subsequently genotyped in a further 497 CBTs with performance data and available blood sample.”

It is well accepted that the DMRT3 genotype is significant to racing performance. However, as shown in the Supplement, 92 of CBT did not have a DMRT3 genotype. How was this handled? It seems quite important to the result.

Author response

Thanks for pointing this out. The majority of these 92 horses (n = 79) lack performance results and only three of them were included in the racing performance analyses. Even so, we have now genotyped the horses for the DMRT3 variant and this data is included in Table S12. However, due to the lack of available DNA, 16 samples were not genotyped. We have re-run the racing performance analysis for SNP rs69244089 without the three horses lacking DMRT3 genotype. Also, we removed one horse from the haplotype racing association analysis, that lacked DMRT3 genotype.

Describe the method (software and parameters) used to phase haplotypes (line 599).

Author response

We have added the following text, line 604:” Prior to association analysis, maximum likelihood estimates were used to determine the 14 SNVs haplotypes with the haplo.em function in the haplo.stat package (103).”

The 14 fine-map variants (as shown in Fig 1B) appear to be quite biased toward the distal end of the 5.5kb region of interest (10 of the 14 within 2500bp). Could the lack of representation of markers earlier in the region be biasing results? With the marker distribution it is not surprising that these markers are in high LD.

Author response

We agree that the LD pattern is not surprising, it is expected. And while the 14 fine mapped variants do cluster at the 3’ end of the region, this is also not unexpected. There are a few factors in play here, and we have added a figure below to aid discussion.

First, our hypothesis, based on historical record, has been that this performance-associated selective sweep i) was introgressed from SBs into CBTs, ii) rose in frequency in CBTs given the selection for performance and iii) is now maintained in CBTs at high frequency but will accumulate new mutations over time. We don’t know the timing of the SB/CBT admixture event, or how many haplotypes were involved, but we would expect high LD for the region given the admixture/selection hypothesis.

In 2018, we identified sweep regions in CBT, likely introgressed from SBs. The original 19.6 kb selective sweep was defined by seven variants. Five of these were strongly associated with performance (1,531 bp span, r2=0.92–0.94), and these were bookended with the next two variable flanking markers. This was so as to account for variants that may have been associated with performance, but that were not available for genotyping using the technology available at the time. This expanded the five SNVs 1.5 kb region to 19.6 kb (Jäderkvist Fegraeus K, *et al*. A potential regulatory region near the EDN3 gene may control both harness racing performance and coat color variation in horses. Physiol Rep. 2018 May;6(10):e13700. doi: 10.14814/phy2.13700). See “2018 Sweep 7 SNVs” figure panel for the positions.

In the current manuscript, we aimed to refine the 19.6 kb region, by first using genotypes from a denser SNV array (from 50K to 670K) and a larger performance phenotyped horse material (629 CBTs). This step did not add the refinement that we needed, so we discovered additional variable positions with WGS, and used segregation analysis to prioritize those positions to be added to a mass array for genotyping. See “77 Variants panel” figure panel for the positions of most of these.

As you can see, the spread of the discovery variants is not even, and is clustered around the five variants strongly associated with performance in 2018. In addition, most of the discovery variants at the 5’ end of the 2018 selective sweep did not match the required segregation pattern and so were not available for selection on the array. The variant positions included in the mass array, and their resultant genotypes are included in Supplementary Table 12. It is of course possible that an untyped flanking variant is also associated with performance, but we have done our utmost to identify the core associated region, and then used functional data to suggest which positions within that region are likely contributors to the complex phenotype of performance.

Is this a typo on line 690: “and not included as standard?” This needs correction/clarification.

Author response

We updated the sentence to now state: “we created the genome library required for motifbreakR as it is not supplied with this package.”
